# Optimizing the layer thickness of sol–gel-derived TiO_2_ coating on polyetheretherketone

**DOI:** 10.1038/s41598-021-95572-9

**Published:** 2021-08-05

**Authors:** Makoto Hayashi, Takayoshi Shimizu, Masashi Imamura, Shunsuke Fujibayashi, Seiji Yamaguchi, Koji Goto, Bungo Otsuki, Toshiyuki Kawai, Yaichiro Okuzu, Shuichi Matsuda

**Affiliations:** 1 Department of Orthopedic Surgery, Kyoto University Graduate School of Medicine, 54 Kawahara-cho, Shogoin, Sakyo, Kyoto, 606-8507 Japan; 2 Medical Device Development Division, Ishihara Sangyo Kaisha, LTD, Osaka, 550-0002 Japan; 3 Department of Biomedical Sciences, College of Life and Health Sciences, Chubu University, Kasugai, Aichi Japan

**Keywords:** Biotechnology, Medical research

## Abstract

Sol–gel-derived TiO_2_ coatings have been confirmed to effectively promote bone-bonding behavior on polyetheretherketone (PEEK) surfaces; however, the optimal layer thickness to maximize the osseointegration and adhesive performance has not been yet determined. In this study, we applied sol–gel-derived TiO_2_ coatings with different layer thicknesses (40 and 120 nm) on PEEK implants to determine the effects of layer thickness on the surface characteristics, adhesive strength, and bone bonding capabilities (including histological osseointegration). The surface analysis results of both coated implants indicated no significant differences concerning the water contact angle, layer adhesion strength, and apatite formation ability in a simulated body fluid. Additionally, the in vivo biomechanical tests revealed a higher bone-bonding strength for both coated PEEK implants (compared with that of the uncoated sample). It was thus concluded that the factor of layer thickness marginally influences the bioactive advantages attained by sol–gel-derived TiO_2_ coatings on PEEK surfaces, highlighting the significant versatility and clinical availability of this coating technology.

## Introduction

Polyetheretherketone (PEEK) is a high-performance thermoplastic polymer that is widely utilized in orthopedic and neurosurgical applications, particularly in spinal fusion devices. One of the primary clinical attributes of PEEK implants when used as intervertebral cages, is their very low elastic modulus that minimizes the subsidence rate and provides a stress shielding effect^1,2^. Another benefit is their radiolucency which significantly facilitates the evaluation of the fusion status during radiographic assessment^3^. Conversely, a severe downside of PEEK hindering its application in spinal implants is its bioinertness, which impedes bone-implant integration and ultimately leads to pseudarthrosis^4,5^. Thus, various techniques to modify the surface properties of PEEK and achieve bone-bonding capabilities have been conducted, including hydroxyapatite (HA) plasma-spraying and titanium coating^2,6–8^.

When deposited on the material surface, sol–gel-derived TiO_2_ coatings can form extremely thin (nano-scale) uniform oxide layers that never degrade^9^. Furthermore, the temperature required for the sol–gel coating process is significantly lower than the above-mentioned coating techniques and, therefore, does not exceed the glass transition temperature of PEEK. In a previous study, we reported on the in vitro and in vivo bioactivity of a sol–gel-derived TiO_2_ layer coated on a PEEK rabbit bone implant model^10^. In that report, however, the thickness of the coating layer was not considered and data regarding its adhesion strength was lacking. For clinical applications, however, the standardization of the coating layer thickness and the assessment of the corresponding adhesive strength are required parameters.

The aim of this study was to determine the optimal thickness for maximizing the bioactivity and adhesion strength of sol–gel-derived TiO_2_ layers on medical PEEK implants and ultimately establish this technology as a standardized clinically available coating method.

## Materials and methods

### TiO_2_ layer coating

The plate-shaped implants (15 × 10 × 2 mm) used for the analyses were cut from a PEEK substrate (TECAPEEK natural, Ensinger Gmbh, Germany: Poisson’s ratio 0.4, specific gravity 1.3, flexural modulus 4.2 GPa, tensile strength 97 MPa). Subsequently, the PEEK implants were subjected to O_2_ plasma treatment and then coated using previously reported sol–gel TiO_2_ coating processes^10^. The implants were placed in the chamber of a vacuum plasma system (YHS-G200SUS, SAKIGAKE-Semiconductor Co., Ltd., Japan) and underwent microwave plasma treatment at 4.8 watts under an O_2_ atmosphere and 10 Pa pressure for 10 min. Following the O_2_ plasma pretreatment, the implants were dipped in the TiO_2_ sol–gel solution consisting of titanium tetraisopropoxide (TTIP), H_2_O, ethanol (EtOH), and nitric acid (HNO_3_) with a TTIP:H_2_O: EtOH:HNO_3_ molar ratio of 1:1:37:0.1. In this study, three different TiO_2_ layer thicknesses were applied to the PEEK implants: 40 nm (T40), 120 nm (T120), and no layer (Uncoated). The layer thickness was controlled by adjusting the concentration of the TiO_2_ sol–gel solution. For the T40 and T120 coatings, the weight ratios were adjusted to 20% and 60%, respectively, with butyl cellosolve and isopropanol. After dipping the implants for 1 min in the solution, they were quickly removed and spin coated at 100 × g (ACT-300A, ACTIVE Co.,Ltd., Japan), followed by air-drying at 80 °C for 24 h. After drying, the implants were soaked in 0.1 M HCl solution at 80 °C for 24 h and then gently washed with ultrapure water. These three steps (O_2_ plasma pretreatment, sol–gel TiO_2_ coating, and acid posttreatment) are a prerequisite for enabling the TiO_2_ layer to solidly adhere to the PEEK substrate and endow it with osseointegrative capabilities.

### Surface characterization

#### Scanning electron microscopy (SEM)

The surface morphology and titanium distribution of the PEEK implants with different coating layer thicknesses were examined via SEM combined with energy dispersive X-ray spectroscopy (EDX) (JSM-7900F; JEOL, Japan) after coating with carbon. The cross-sectional and surface morphologies of the PEEK implants were examined by SEM (S-4800; Hitachi Ltd, Tokyo, Japan) after coating with Pt/Pd.

#### Water contact angle

The hydrophobic characteristics of the PEEK implants were determined by measuring the water contact angle via an automated contact angle meter (DMo-501, Kyowa Interface Science Co., Ltd, Japan).

#### Surface roughness (micrometer scale)

A contact probe profilometer (Mitutoyo Surftest SV-2000) was used to measure the surface topography of the coatings on the micrometer scale. Initially, the surface roughness (Ra) was measured in the direction perpendicular to the polished direction at five different areas of each implant. The average Ra was calculated from these five areas.

#### Apatite-forming ability of the implant surfaces

The apatite-forming properties of the samples were examined by soaking them in a simulated body fluid (SBF) at pH 7.40 for 3 days at 36.5 °C. Soaking at 36.5 ± 0.5 °C is recommended by the ISO 23,317, and SBF has been verified to generate reproducible result. The ion concentrations (all in mM) were as follows: Na^+^, 142.0; K^+^, 5.0; Ca^2+^, 2.5; Mg^2+^, 1.5; Cl^−^, 147.8; HCO^3−^, 4.2; HPO_4_^2−^, 1.0; SO_4_^2−^, 0.5. The samples were removed from the SBF, washed with distilled water, and dried on a clean bench. Their surfaces were examined via SEM and their apatite formation performances were determined by the presence of spherulites consisting of tiny flake-like crystals, which is the characteristic morphology of SBF-deposited apatite species.

#### Adhesive strength testing

The adhesive strength was examined using the ASTM (American Society for Testing and Materials) standard method for the tension test, as well as the ISO standard method for the tape test. In the ASTM F-1147 test, the failure load / area (MPa) was measured^11^. The tape test was performed using a universal strength tester (EZ-Graph, SHIMADZU CORPORATION, Japan) in accordance with the ISO 2409.

### In vivo* study*

#### Animals and surgical procedure

This study was approved by the Animal Research Committee of the Graduate School of Medicine, Kyoto University (Approval number #Medkyo 21251). Furthermore, all procedures were performed in accordance with the regulations and guidelines for animal experimentation provided by this committee. We complied with the ARRIVE guidelines for reporting animal experiments. Fifty mature male Japanese white rabbits (weight: 2.8–3.5 kg) were used. Twenty seven of the 50 rabbits were for biomechanical testing, and the remaining 23 were for histological and radiological analysis. The animal tests were conducted according to three experimental time-points (4-, 8-, and 12-week groups), with nine animals (18 legs) in each group for the biomechanical evaluation of the three implants (uncoated, T40, T120) (n = 6), and eight animals (16 legs) per group for the histological and radiological analyses of the three implants (n = 5).

The PEEK implants were sterilized with ethylene oxide gas. The surgical methods used have been described previously^10^. The rabbits were anesthetized with an intravenous injection of pentobarbital sodium (40 mg/kg), an inhalation of isoflurane, and local administration of 1% lidocaine solution. A 3-cm longitudinal skin incision was made on the medial side of the knee, and the fascia and the periosteum were incised and retracted to expose the tibial cortex. A 16 × 2 mm slit-like perforation was cut using a dental bur from the medial to the lateral cortex within the proximal metaphysis of the tibiae, parallel to the longitudinal axis of the tibiae. After the hole was irrigated with saline, each coated PEEK plate was implanted (Fig. 2a). Following the installation of the implants, the animals were housed individually in standard rabbit cages.

After 4, 8, and 12 weeks post-operation, the rabbits were sacrificed with intravenous pentobarbital sodium. The segments of the proximal tibial metaphyses containing the implanted plates were cut and prepared for the biomechanical tests. All specimens were kept moist after harvesting. To remove the periosteal bone growth, the bone tissue surrounding the plates was carefully removed from both sides and the ends using a dental bur.

#### Biomechanical testing

The detaching tests were performed within 24 h of explantation following the protocol procedures. Traction was applied vertically to the implant surface at a cross-head speed of 35 mm/min using an Instron-type autograph (Model 1011; Aikoh Engineering Co. Ltd., Nagoya, Japan) with specifically designed hooks to hold the bone-plate-bone construct (Fig. 2a). The detaching failure load was measured when the plate detached from the bone. If the plate detached before the test, the failure load was defined as 0 N.

#### Radiological analysis

After harvesting the tibia from the rabbits, a $\upmu$-CT scan (SMX-100CT-SV-3; Shimadzu Corp., Kyoto, Japan) with a slice thickness of 0.04 mm was taken. Three-dimensional images of the harvested bone containing the PEEK implants were reconstructed using a software package provided by the manufacturer (VG studio MAX 2.2, Volume Graphics GmbH, Heidelberg, Germany). To measure the new bone volume (NBV) surrounding the implants, the region of interest was determined as a sphere with a radius of 2.5 mm within the cancellous bone of the same tolerance and condition. The NBV was defined as the region with the same density as the cortical bone within the sphere.

#### Histological analysis

After the $\upmu$-CT scan, the specimens were fixed in a 10% phosphate-buffered formalin fixative (pH 7.25) for 10 d, then dehydrated in serial concentrations of ethanol (70%, 80%, 90%, 99%, 100%, and 100% [v/v]) for 3 d at each concentration, and finally embedded in polyester resin. Next, thick sections (500 µm) were cut perpendicular to the tibial axis using a band saw (BS-3000CP, EXACT cutting system; Exakt Apparatebau GmbH, Norderstedt, Germany) and then ground to a thickness of 80 µm using a grinding-sliding machine (Microgrinding MG-4000; Exakt Apparatebau GmbH). Each section was then stained with Stevenel’s blue and van Gieson’s picrofuchsin, which differentially stained calcified bone bright red (with the intensity depending on the maturity of the bone), non-calcified bone and osteoid green, and soft tissue blue. Thorough microscopic analysis was performed on the histological slides using a transmitted light microscope (Eclipse 80i; Nikon, Tokyo, Japan) with a digital camera (DS-55M-L1; Nikon, Tokyo, Japan). The stained sections were examined by quantitative histomorphometry to evaluate the amount of bone tissue directly in contact with the PEEK implant surface, dubbed as the bone implant contact (BIC), using the 2-dimensional (2-D) image processing software (Image J; National Institutes of Health, Bethesda, MA, USA). The BIC was calculated after the tissue-implant contact area had been manually defined. The specimen sections were also evaluated using SEM.

### Statistical analysis

Four samples of each of the three implant groups were used for each experiment (n = 5), except for biomechanical testing, where six of each of the three implant types were tested (n = 6). Statistically significant differences between the three groups were measured using one-way analysis of variance (ANOVA) and Tukey’s multiple comparison tests. *p* < 0.05 was considered significant. All the statistical analysis was performed using JMP software (version 8.02, SAS Institute, Cary, NC, USA).

## Results

### Surface characteristics

**Figure 1:**
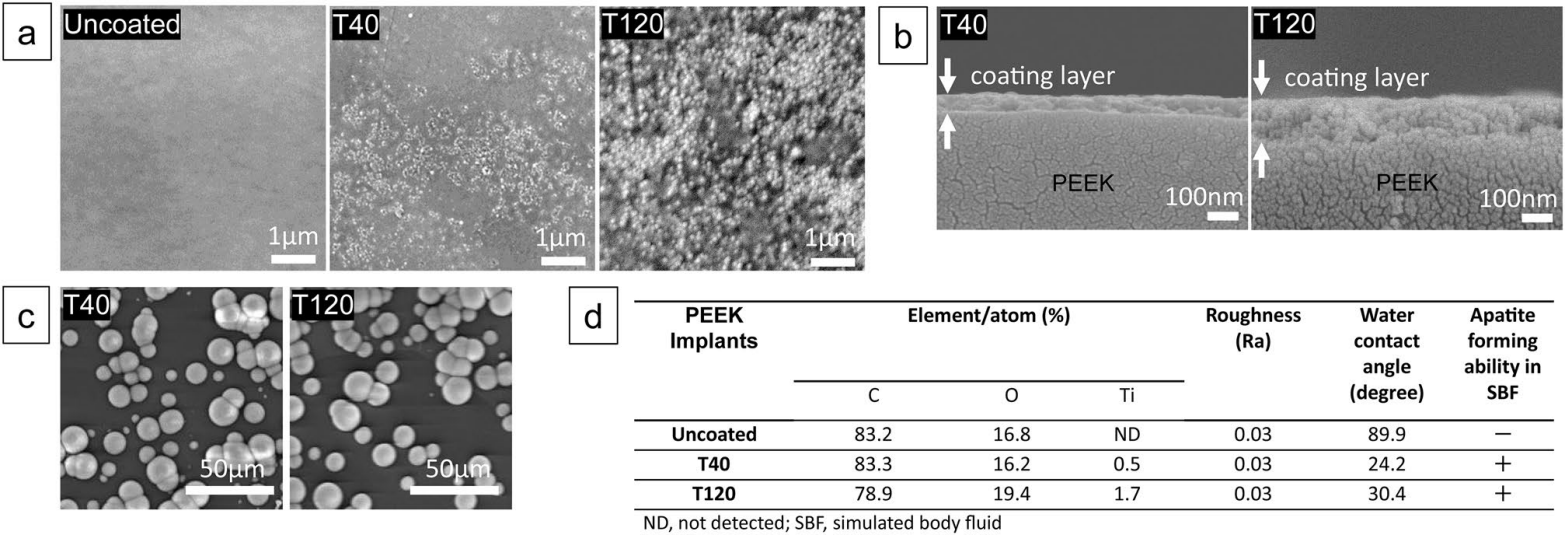
SEM images and surface characteristics of the PEEK implants: (**a**) uncoated implant shows a smooth surface, whereas the T40 and T120 implants show nano-scale precipitation on their surface; (**b**) cross-sectional SEM images of the T40 and T120 implants showing the uniformity of the sol–gel TiO_2_ coating layers; (**c**) SEM images three days after soaking in a SBF demonstrating spherical apatite formation on the surface; (**d**) summary of the surface characteristics (Ti elements detection, uniform surface roughness, improved hydrophilicity) of the T40 and T120 implants.

Figure 1 illustrates the surface characteristics of the PEEK implants with different coating layer thicknesses. Figure 1a shows the surface SEM images of the implants: the uncoated implant with smooth surface, the T40 implant with precipitated nanometer-fine TiO_2_ particles, and the T120 implant with more evident nano-particles appeared on the surface. The cross-section of the coated implants (T40 and T120) revealed a uniform TiO_2_ layer covering the PEEK substrate for both instances (Fig. 1b). Figure 1c shows the SEM images of the implant surfaces after soaking in SBF: spherical apatite formation with clearly detectable calcium phosphate elements (identified by EDX analysis) was observed for both the T40 and T120 samples, indicating that this apatite layer could serve as a foundation for the in vivo direct bone bonding process. Figure 1d summarizes the surface characteristics results of each implant: EDX analysis, surface roughness, wettability, and apatite forming ability in SBF. Although the measured micro-scale roughness did not significantly differ between the three implants, the water contact angle was significantly improved in the T40 and T120 samples.

According to the ASTM standard adhesive strength tests, the failure load was 0.49 ± 0.11 MPa and 0.51 ± 0.04 MPa for the T40 and T120 implants, respectively. The failure was observed between the adhesive apparatus and the coating layer, while the TiO_2_ layer remained on the PEEK substrate. Based on the tape tests, the critical normal load (Lc), which indicates the load levels necessary to cause identifiable coating disruption, exceeded 40 N for both the T40 and T120 implants.

### In vivo* study*

#### Biomechanical behaviors

**Figure 2:**
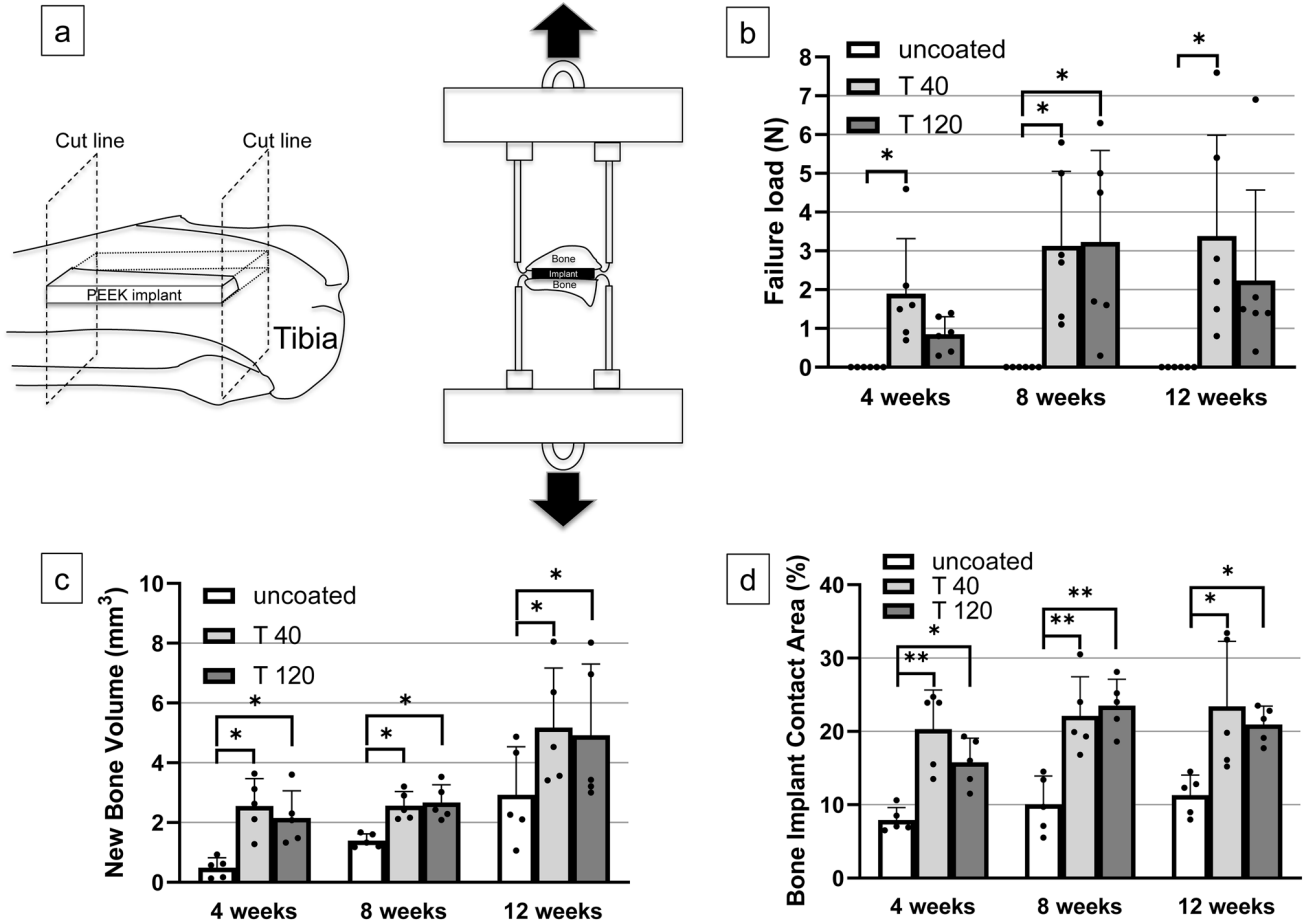
Results of the in vivo analyses of the rabbit tibia: (**a**) scheme of the detaching test measurements. The PEEK plates were implanted into the rabbit tibia and the bone-implant bonding strength was measured after harvesting; (**b**) failure load results for each study period; (**c**) new bone formation volume surrounding the PEEK implants according to the three-dimensional μCT analysis; (**d**) bone-implant contact ratio during the histological analysis (**p < 0.01, *p < 0.05).

Figure 2b shows the results of the detaching tests. At 4 weeks after implantation, the average failure load of the T40 implant was greater than that of the uncoated implant (*p* = 0.004), suggesting a superior bone-implant bonding strength for the former. After 8 weeks, the average failure load of the T40 and T120 implants was greater compared to that of the uncoated implant (*p* = 0.019 and 0.015, respectively). After 12 weeks, the average failure load of T40 was greater than that of the uncoated implant (*p* = 0.027).

#### New bone formation

**Figure 3:**
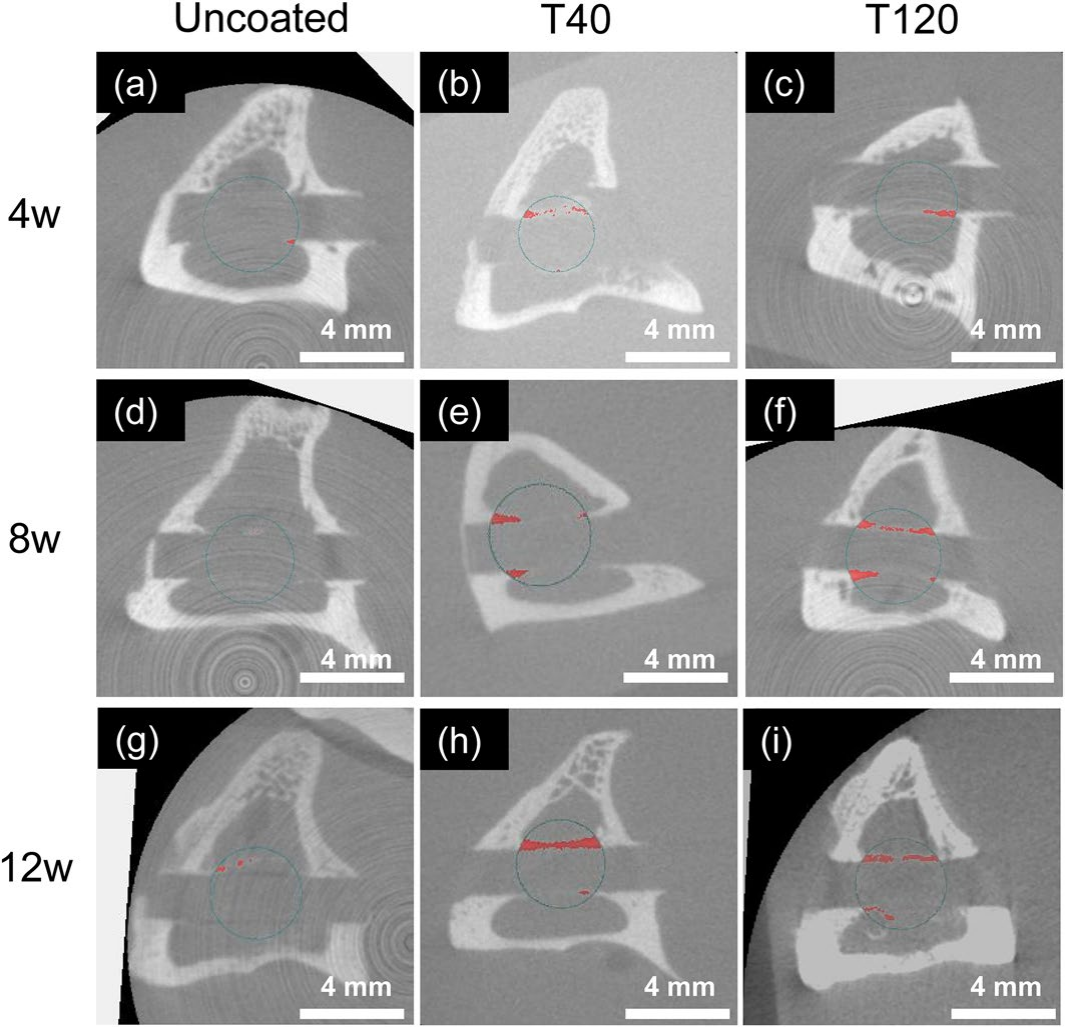
In vivo μCT images. The new bone volume surrounding the PEEK surfaces (red) inside the sphere (radius 2.5 mm) within the cancellous bone (green circle) was measured: (**a**–**c**) limited new bone formation was observed after 4 weeks; (**d**–**f**) T120 presented greater new bone formation compared to the uncoated implant after 8 weeks; (**g**–**i**) both T40 and T120 showed rich new bone formation, as opposed to the uncoated implant after 12 weeks.

Figure 3 shows the µ-CT cross-sectional images revealing the new bone formation surrounding the PEEK implants: the uncoated implant exhibited scarce bone formation on its surface throughout all study periods, whereas the T40 and T120 implants demonstrated remarkable osteoconductive behavior on their surfaces. The three-dimensional bone volume analysis verified that these differences were statistically significant after 4 and 8 weeks (T40: *p* = 0.002 [4-week] and 0.004 [8-week]; T120: *p* = 0.012 [4-week] and 0.002 [8-week]) (Fig. 2c).

#### Histology and histomorphometry

**Figure 4:**
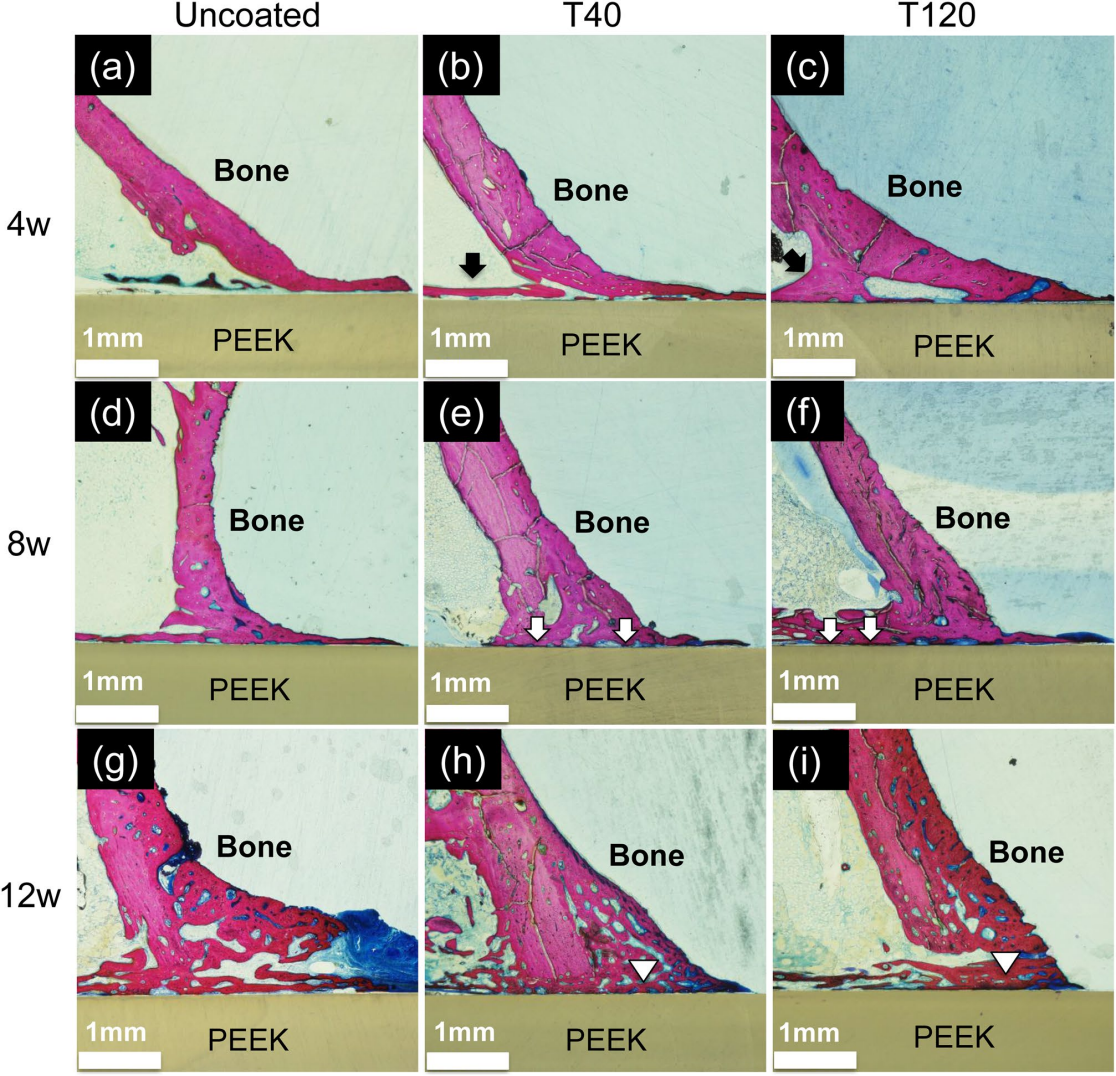
Histological images: (**a**) new bone formation (black arrows) was observed surrounding the T40 and T120 implants at 4 weeks, contrary to the uncoated implant. At 8 weeks, T40 and T120 exhibited direct contact between the new bone tissue and the PEEK surface (white arrows). At 12 weeks, the new bone had evolved into mature bone in the T40 and T120 implants, and extended contact with the PEEK surface was observed (white arrowheads).

Figure 4 shows the results of histological analysis. At 4 weeks, immature bone formation was present around the old bone tissue in the T40 and T120 implants. At 8 weeks, the newly formed bone tissue began to mature (but not in the uncoated implant); parallelly, direct contact between the new bone tissue and the implant was observed. At 12 weeks, even more mature lamellar bone tissue was observed to be directly attached on the surfaces of the T40 and T120 implants, whereas a gap was observed in the uncoated implant. Direct contact was rarely observed in the uncoated implant throughout the experiment period.

**Figure 5:**
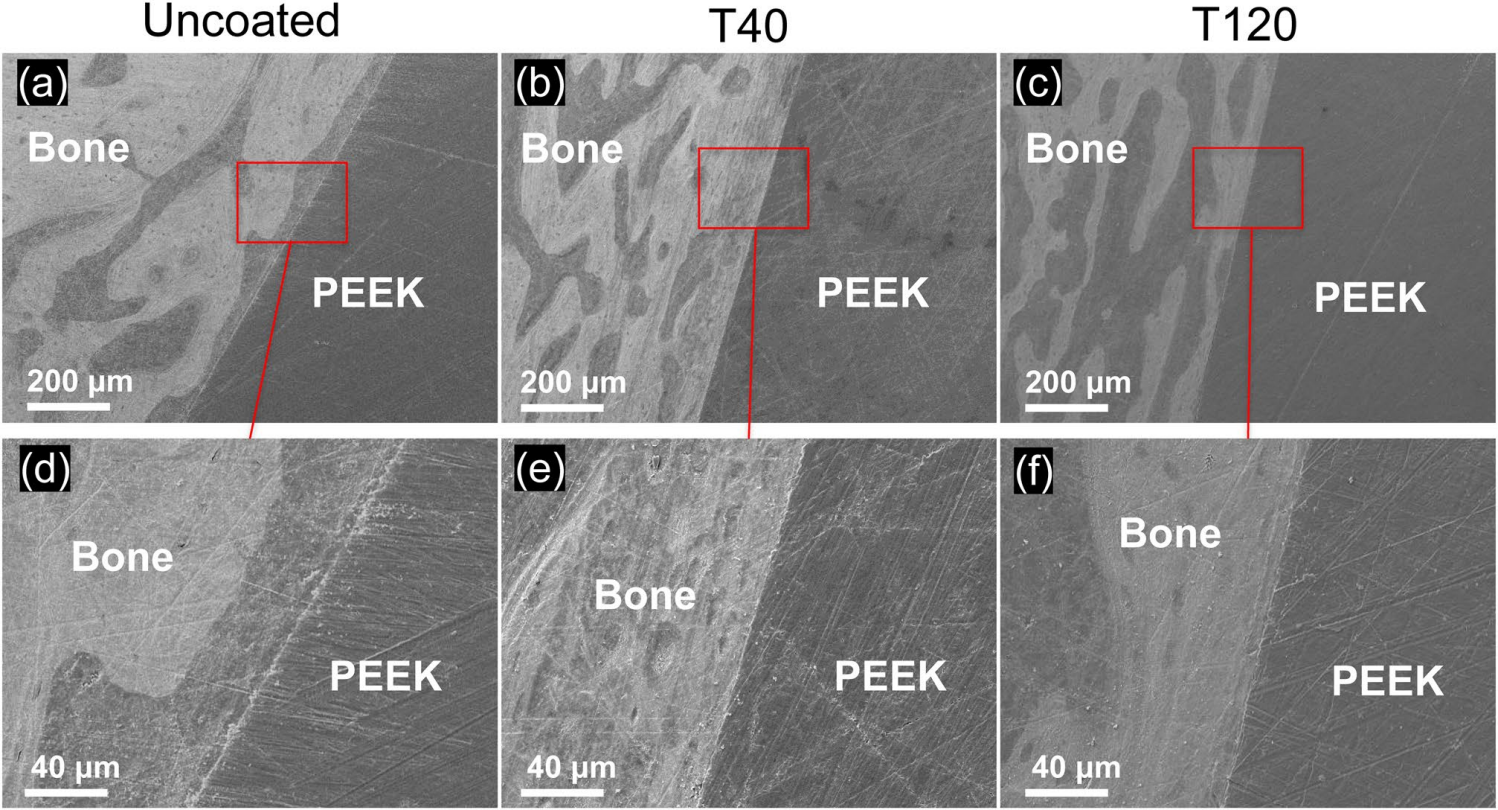
SEM images of the histological section at 12 weeks: (**a**,**d**) uncoated implant (direct bone-implant contact was scarcely observed); (**b**,**e**) T40 implant (bone-implant integration was observed); (**c**,**f**) T120 implant (bone-implant integration was observed).

The BIC analysis revealed that the T40 and T120 implants demonstrated a more enhanced BIC than the uncoated implant after 4, 8, and 12 weeks (T40: *p* = 0.001 [4-week], 0.002 [8-week], 0.012 [12-week]; T120: *p* = 0.016 [4-week], 0.001 [8-week], 0.043 [12-week]) (Fig. 2d). The SEM images of the histological sections after 12 weeks (Fig. 5) confirmed that direct bone-implant integration had manifested in the T40 and T120 implants: in the uncoated implant (Fig. 5a,d), direct bone-implant contact was rarely observed (interfered with connective tissue), whereas the T40 and T120 implants demonstrated direct bone-implant integration (Fig. 5b,c and c,e respectively).

## Discussion

After approval by the United States Food and Drug Administration in 1998, PEEK has been widely applied as a spinal fusion device component. In particular, PEEK can significantly reduce the risk of cage subsidence, especially when used as a spinal interbody cage, owing to its low elastic modulus (close to the human cortical bone)^12^. In addition, its radiolucent property renders the evaluation of the postoperative fusion status by a plain radiograph or CT scan a considerably easier task^3^. Despite its clinically favorable characteristics, the surface bioinertness of PEEK may induce inflammatory cell infiltration and subsequent fibrous tissue formation, ultimately resulting in pseudarthrosis^5,13^. To address this complication, in our previous work we developed a sol–gel-derived TiO_2_ coating for application on PEEK surfaces^10^. This coating method can achieve a nano-scale thin and uniform coating layer and is based on a simple and cost-effective process. More importantly, the temperature required for the coating process is significantly low (maximum 80 °C), thereby remaining below the glass transition temperature of PEEK (approximately 143 °C). Our initial report, however, did not include detailed data regarding the thickness of the coating layer, as well as the minimum adhesive strength required for clinical application. Thus, in the present study, we prepared PEEK implants with two different coating layer thicknesses (T40: 40 nm and T120: 120 nm) and compared their surface characteristics, adhesive strength, and bone bonding capabilities (including histological osseointegration). Our results demonstrated no significant differences in regard to the surface characteristics between the two thickness layers. In terms of the adhesive strengths, the load levels necessary to cause identifiable coating disruption exceeded the measurement limit for both the T40 and T120 implants. The *in-vivo* biomechanical tests and histological analysis results confirmed the similar bone-bonding abilities of the two thickness layers. These results implicate that, regardless of the layer thickness, sol–gel-derived TiO_2_ coatings can endow PEEK implants with bioactive properties, highlighting the high versatility and clinical availability of this coating technology.

In our original report, the sol–gel TiO_2_ layer was roughly 30–50 nm and, therefore, we set 40 nm as the target thickness in this study. Furthermore, we evaluated the characteristics of 80 nm- thick coating layer in the preliminary experiment and found that there is no specific difference between 80 nm-thick and 120 nm-thick coating layers in terms of their in vivo bone bonding ability. The 40 nm-thick coating layer entails a more facile and cost-effective coating process than its 120-nm counterpart. In addition, a thinner layer allows for the preservation of the surface morphology, especially when the substrate exhibits nano-scale roughness. Accordingly, we recommend employing the 40-nm thickness for implant manufacturing.

The process of the sol–gel TiO_2_ coating derives from the following chemical theory: O–C=O, C=O, and C–O groups are generated on the PEEK surface after the O_2_ plasma pretreatment stage, and they are characterized by distinctive affinity with the Ti–O in the TiO_2_ gel^14^. Additionally, the surface hydrophilicity is increased following the TiO_2_ coating (Fig. 1d), which is considered to be favorable for cell adhesion. Acid post-treatment is necessary to provide the TiO_2_ layer with apatite-forming properties in a SBF. The crystalline phase of TiO_2_ is important owing to its performance^15,16^ and can be changed during the treatment process. Kokubo et al. found that the sol–gel derived TiO_2_ layer exhibited an amorphous structure at pre-acid treatment stage, and then changed into a positively charged crystalline brookite with a little amount of rutile and anatase (i.e., post-acid treatment)^17^. This positive charge of the surface is a key factor for the apatite formation properties of the TiO_2_ layer in a SBF; the positive charge of the surface binds to the negative charge of the phosphate ions in the SBF, causing the absorption of positively charged calcium ions, and eventually leading to apatite formation^18^. The apatite-forming capabilities of SBF can be used as a reliable predictor for the in vivo bone-bonding performance. When the apatite layer is generated in a physiological condition, it exhibits extremely similar composition and structure with bone minerals compared with artificially sintered HA^19^ and plays a role as a foundation substrate for the direct bone bonding process. In the current analysis, we discovered that the thickness of the layer does not affect the apatite forming properties of sol–gel-derived TiO_2_ coatings (Fig. 1c).

The adhesive strength of the layer was tested using the two standard methods. During the ASTM standard test, the failure occurred between the adhesive apparatus and the coating layer, and the TiO_2_ layer remained on the PEEK substrate. This behavior indicated that the adhesive strength of both the T40 and T120 implants satisfied the standardized requirements for clinical application. The tape test constitutes another standard method for evaluating the adhesion strength, and in our work, revealed an adhesive ability exceeding 40 N for both T40 and T120. These findings indicate that the layer thickness does not have significant impact on the adhesive strength of the sol–gel TiO_2_ coating layer. As long as the layer thickness remains nano-scale thin, the adhesive strength can be ensured.

According to the biomechanical tests and histological evaluations, both sol–gel TiO_2_ coated implants demonstrated significantly higher bone-implant integration compared with the uncoated implant. The average failure load at 12 weeks (3.0 N) in the current analysis was relatively lower than that of our initial study (average 15–20 N). This is theoretically attributed to the low surface roughness (Ra = 0.03) of the PEEK implants used in this study. The PEEK implants in the initial study had a rougher surface (Ra = 0.14), since they were polished with a 800 grit SiC paper before applying the sol–gel TiO_2_ coating. Polishing trace induces micro-scale roughness on the PEEK surface, which can promote the bone-bonding ability^20,21^. To further facilitate the bone-implant integration for clinical use, the surface roughness underneath the coating layer should also be considered. The osteoconductive effect of the coating layer, which was confirmed in the CT scan analysis, had no significant dependence on the layer thickness. This phenomenon indicates that the in vivo influence of the coating layer thickness is minimal as well.

## Conclusions

In the present study, we analyzed sol–gel-derived TiO_2_ coatings with two different layer thicknesses (T40: 40 nm and T120: 120 nm) and compared their surface characteristics, adhesive strength, and bone bonding abilities (including histological osseointegration). The surface characteristics analysis showed no significant differences concerning the water contact angle and apatite formation performance in a simulated body fluid between the two layer thicknesses. More importantly, both coating layers exhibited comparable adhesive strengths, and the in vivo biomechanical tests revealed higher bone-bonding strengths for the PEEK implants under both coating layer thicknesses, compared with that of the uncoated sample. Summarizing, sol–gel-derived TiO_2_ coatings on PEEK implants can effectively advance bone-growth during the post-operation stage, regardless of the coating layer thickness. We hope for our findings to actively contribute in the establishment of a highly versatile and clinically standardized coating technology for advanced medical implants.
